# P2Y6 receptor inhibition aggravates ischemic brain injury by reducing microglial phagocytosis

**DOI:** 10.1111/cns.13296

**Published:** 2020-03-10

**Authors:** Ruo‐Xue Wen, Hui Shen, Shu‐Xian Huang, Li‐Ping Wang, Zong‐Wei Li, Peng Peng, Muyassar Mamtilahun, Yao‐Hui Tang, Fan‐Xia Shen, Heng‐Li Tian, Guo‐Yuan Yang, Zhi‐Jun Zhang

**Affiliations:** ^1^ Shanghai JiaoTong Affiliated Sixth People's Hospital School of Biomedical Engineering Shanghai Jiao Tong University Shanghai China; ^2^ Department of Neurology School of Medicine Ruijin Hospital Shanghai Jiao Tong University Shanghai China; ^3^ Department of Neurosurgery Shanghai Jiao Tong University Affiliated Sixth People's Hospital Shanghai Jiao Tong University Shanghai China

**Keywords:** ischemia, microglia, P2Y6 receptor, phagocytosis

## Abstract

**Introduction:**

Clearance of damaged cells and debris is beneficial for the functional recovery after ischemic brain injury. However, the specific phagocytic receptor that mediates microglial phagocytosis after ischemic stroke is unknown.

**Aim:**

To investigate whether P2Y6 receptor‐mediated microglial phagocytosis is beneficial for the debris clearance and functional recovery after ischemic stroke.

**Results:**

The expression of the P2Y6 receptor in microglia increased within 3 days after transient middle cerebral artery occlusion. Inhibition of microglial phagocytosis by the selective inhibitor MRS2578 enlarged the brain atrophy and edema volume after ischemic stroke, subsequently aggravated neurological function as measured by modified neurological severity scores and Grid walking test. MRS2578 treatment had no effect on the expression of IL‐1α, IL‐1β, IL‐6, IL‐10, TNF‐α, TGF‐β, and MPO after ischemic stroke. Finally, we found that the expression of myosin light chain kinase decreased after microglial phagocytosis inhibition in the ischemic mouse brain, which suggested that myosin light chain kinase was involved in P2Y6 receptor‐mediated phagocytosis.

**Conclusion:**

Our results indicate that P2Y6 receptor‐mediated microglial phagocytosis plays a beneficial role during the acute stage of ischemic stroke, which can be a therapeutic target for ischemic stroke.

## 1 INTRODUCTION

Ischemic stroke is one of the leading causes of death and adult disability around the world, which leads to massive cell death and complex pathological changes.^1^, ^2^ Microglial activation is one of the characteristics at the acute stage of stroke.^3^ Microglia, the innate immune cells in the central nervous system, firstly respond to the injury and are recruited to the infarct area after ischemic stroke.^4^, ^5^

Activated microglia can enhance proliferation and phagocytosis. It functions by releasing a series of proinflammatory or anti‐inflammatory cytokines depending on the different phenotypes.^6^, ^7^ On one hand, activated microglia can secrete a series of proinflammatory cytokines to aggravate the destruction of the blood‐brain barrier and tissue damage.^3^, ^8^ On the other hand, they promote tissue repair by phagocytosis or secreting neurotrophic factors.^9^ Approaches to amplify the beneficial role of microglia may promote neurological function recovery after stroke.

The fast engulfment and clearance of cell debris or dead cells are necessary for the nervous system remodeling and neurological function recovery after brain injury.^10^ Microglia are important cells that engulf damaged cells for tissue repair after brain injury.^11^, ^12^ CD36, CX3CR1, TLR4, TREM2, MerTK, and MFG‐E8 have been shown to mediate microglial phagocytosis after ischemic stroke.^13^, ^14^, ^15^, ^16^, ^17^ CD36 knockout neonatal mice displayed an enlarged area of brain infarct at the first day after stroke, and TREM2 knockout mice showed larger infarct size following more serious neurological impairment at 14th day after stroke.^13^, ^14^ However, MerTK knockout and MFG‐E8 knockout mice on 28th day after ischemic stroke showed reduced the brain infarct area.^16^ The evidence demonstrated that microglial phagocytosis has both beneficial and harmful properties, which depend on the specific receptors and the time point after brain injury.

P2Y6 receptor is expressed on microglia and can be activated by the endogenous agonist UDP leaking from injured neurons to mediate microglial phagocytosis.^18^ Inhibition P2Y6 receptor exacerbated brain injury after radiation by blocking microglial phagocytosis in mice.^19^ Another study showed that blocking the P2Y6 receptor could prevent LPS‐induced cell death in vivo and in vitro.^20^ Similarly, the microglial P2Y1 receptor, which can be activated by extracellular ATP, mediate the neuroprotective effects during ischemic stress and OGD insult.^21^ All these evidence intrigued us to hypothesize that P2Y6 receptor is a key mediator of microglia phagocytose during the acute phase of ischemic stroke. We found that P2Y6 receptor‐mediated microglial phagocytosis was beneficial for the debris clearance and functional recovery after ischemic stroke.

## 2 METHODS

### 2.1 Experimental design

Animal procedures were approved by the Institutional Animal Care and Use Committee (IACUC) of Shanghai Jiao Tong University, Shanghai, China. All procedures were executed to minimize the pain of animals following protocols. Adult male ICR mice weighing 30‐32 g were randomly divided into three groups: 1) sham (n = 6); 2) saline+ DMSO treated (n = 15); and 3) P2Y6 receptor inhibitor MRS2578 treated (n = 12). Mice were treated with saline or MRS2578 (3 mg/kg) through intraperitoneal injection once a day for 3 days after transient middle cerebral artery occlusion MCAO (tMCAO). Neurological functions were assessed by modified neurological severity scores (mNSS) at 1, 3, 7, and 14 days after tMCAO. Grid walking test were assessed at 3, 7, and 14 days. The mice were sacrificed at 14 days after tMCAO.

### 2.2 Cell culture

Primary microglia and neurons were isolated according to previously described methods.^22^, ^23^ All cells were maintained in a humidified incubator containing 5% CO_2_ at 37°C. Briefly, mixed glial cells were harvested from neonatal Sprague Dawley rat (SD, JSJ) cortexes. The mixed glial cells were cultured for 10‐14 days in Dulbecco's modified Eagle's medium (DMEM; Gibco Laboratories) with 10% fetal bovine serum (FBS; Life Technologies). After the mixed glial cultures reached confluency, primary microglia were isolated from the culture by a brief duration of shaking. Primary microglia were seeded into 24‐well culture plates at a density of 1 × 10^5^ cells per well and were cultured for 24 hours. The microglia were used for phagocytosis assays and coculture.

For neuronal culture, primary cells were harvested from neonatal Sprague Dawley rat cortexes and seeded into a 6‐well hanging cell culture insert at a density of 6 × 10^5^ cells per well. The neurons were cultured in neurobasal‐A medium supplemented with 2% B27 for 5 days and cocultured with primary microglia.

### 2.3 Oxygen‐glucose deprivation

Microglia and neuron cocultures were subjected to oxygen‐glucose deprivation (OGD) for 1 hour and reoxygenation for 11 hours. Cocultures were rinsed twice by PBS and cultured in glucose‐free DMEM. Then, the cells were placed into a specialized anaerobic chamber containing 95% N_2_ and 5% CO_2_ at 37°C for 1 hour to produce OGD. After that, the media was replaced with normal DMEM medium containing 10% fetal bovine serum and neurobasal‐A medium supplemented with 2% B27 containing glucose under normoxic conditions for an additional 23 hours at 37°C as OGD/RP. The cells of the control group were rinsed twice by PBS and cultured in normal neuron and microglia mixed medium under normoxic conditions for 24 hours at 37°C. The cells were harvested for subsequent experiments. All procedures were performed according to the manufacturer's protocol.

### 2.4 Cell proliferation and cytotoxicity assay

Microglia were seeded in 96‐well plates at a density of 2 × 10^4^ cells per well in 100 μL of culture medium with or without 1 μmol/L, 2 μmol/L, or 5 μmol/L MRS2578 (ApexBio). Cells were cultured in a incubator at 37°C for 24 hours. 10 μL of CCK‐8 solution was added to each well. The absorbance at 450 nm was measured by a microplate reader after cell incubation at 37°C for 1‐4 hours.

### 2.5 Phagocytosis assay

Fluorescent latex beads (diameter 2 µm) were purchased from Sigma‐Aldrich. After 12 hours of incubation with 200 ng/mL LPS, 100 μmol/L UDP stimulation, with or without different MRS2578 concentrations treatment, microglia were incubated in conditioned medium with fluorescent latex beads for 2 hours. The cultures were washed with PBS at least three times and fixed by 4% paraformaldehyde. The experiments were repeated independently three times, with three wells in each group. Five visual fields in each well were taken pictures, counted and statistically analyzed. For flow cytometry, after 2 hours incubation with beads, cells were washed with PBS and incubated with antibodies for 20 minutes at 4°C, then subjected to FACScan analysis.

### 2.6 Antagonist administration

The selective P2Y6 receptor antagonist MRS2578 (#B2167) was from ApexBio. The powder was dissolved in DMSO and the storage concentration was 10 mg/mL. Mice were injected intraperitoneally with MRS2578 (3 mg/kg).^24^ Mice were injected with either 3% storage solution diluted in saline or the same concentration of DMSO diluted in saline every day for 3 days. In vitro, MRS2578 (1 μmol/L; ApexBio) was added in primary microglia culture with 200 ng/mL LPS and 100 μmol/L UDP for 12 hours.

### 2.7 Transient middle cerebral artery occlusion (tMCAO) in mice

Transient MCAO surgery was performed as described previously.^25^, ^26^ Mice were anesthetized with isoflurane (1.5‐2%) and oxygen/nitrous oxide (30%/70%). Firstly, an incision was made in the middle of the neck, and the external carotid artery, internal carotid artery and left common carotid artery were isolated carefully by microscopic curved tweezers. A 6‐0 nylon monofilament suture (Dermalon, 1756‐31, Covidien) coated with silicon was gently inserted from the external carotid artery to the internal carotid artery. The insertion stopped until it blocked the middle cerebral artery. The insertion depth was about 0.8‐1 cm. After 90 minutes of ischemia, the suture was withdrawn gently to allow reperfusion. Successful occlusion and reperfusion of the MCA were confirmed by laser Doppler flowmetry (Moor Instruments). Regional blood flow declined more than 80% compared with the baseline and recovered to 80% of the baseline is considered as a successful model.

### 2.8 Neurobehavioral tests

Neurobehavioral tests were performed by an investigator who was blinded to the experimental design. mNSS system ranging from 0 to 14 was used. mNSS is a comprehensive evaluation system of neurobehavioral function including motor, sensory, balance, and reflex tests. Zero represents normal and 14 represents the most severe neurological deficiency.^27^ The bend and torsion condition of suffered limbs were examined by lifting the tails of the mice for the motor test (0‐3). The capacity to walk in a straight line was also observed (0‐3). Neurological deficiencies in balance were evaluated by a balance beam test. Limbs dropping from the beam and ability to successfully cross the beam were evaluated. (0‐6). Corneal and pinna reflex were examined for the sensory and reflex tests (0‐2). The score indicated the degree of neurological deficiency.

Grid walking test was used to measure motor coordination deficits after ischemic stroke for each group.^28^ The mice were placed on an elevated square grid with each grid cell being 1.5 × 1.5 cm^2^. A camera was located below the grid to record the number of foot faults made by the contralateral and ipsilateral limbs. Each time a limb slipped through a grid, a “foot fault” is recorded. Before the formal experiment, the mice were allowed to habituate to the environment for 1 minute. 70% ethanol was used to clean the grid after each trial. Healthy mice are able to walk accurately with their limbs on a grid or made faults symmetrically. Animals with brain damage make more ipsilateral foot faults than healthy animals while passing through the grid. We measured the motor coordination deficits by the ratio: ipsilateral foot faults/(ipsilateral foot faults + contralateral foot faults).

### 2.9 Measurements of brain infarct and atrophy volume

The mice were sacrificed at 3 days and 14 days after tMCAO, and transcardially perfused with the 0.9% saline, followed by 4% paraformaldehyde. For samples gathered from mice sacrificed after day 3, the brains were directly frozen in −80°C for 1 day and cut into 20 μm sections and mounted on slides, from the anterior commissure to the hippocampus. For samples gathered from mice sacrificed after day 14, the brains were postfixed in 4% paraformaldehyde overnight and dehydrated in 30% sucrose until the brain sank to the bottom of the sucrose solution. Then, the brains were quickly frozen in −80℃, cut into 30 μm sections and stored in an antifreeze solution. Sections were used for cresyl violet staining. The infarct area was measured by Image *J* software (NIH). Infarct size was corrected for edema by using the formula: ΔS = contralateral area‐normal area of the ipsilateral hemisphere. Infarct volume between two adjacent sections was calculated by the formula:

$V=\sum_{1}^{n}\frac{H\left[\Delta Sn+\Delta S(n+1)+\sqrt{\Delta Sn+\Delta S(n+1)}\right]}{3}$, in which *H* was the distance between two adjacent sections (*H* = 200 μm) and Δ*Sn* and ΔS*n*+1 were the infarct areas of two adjacent sections. The atrophy volume was calculated with the formula: $=\sum_{1}^{n}\frac{H\left[\Delta Sn+\Delta S(n+1)+\sqrt{\Delta Sn+\Delta S(n+1)}\right]}{3}$, in which *H* was the distance between two adjacent sections (*H* = 300 μm), Δ*Sn* and ΔSn+1 were the atrophy areas of two adjacent sections.

### 2.10 Western blotting analysis

The ischemic brain tissue was sonicated in the protein lysis buffer (RIPA, protease cocktail inhibitor and phosphatase inhibitor). After ultrasonic treatment, the supernatant was collected by centrifugation at 12 000 *g*. The protein concentrations were examined by a BCA kit (Thermo Scientific). An equal amount of protein was loaded onto 10% resolving gel for electrophoresis. Then, the protein was transferred onto a transfer membrane (Merck KGaA). The transfer membranes were blocked by 5% nonfat milk and incubated with the primary antibodies against P2Y6 receptor (1:1000 dilution, Alomone), MLCK (1:1000, Abgent), and β‐actin (1:1000 dilution, Invitrogen) at 4°C overnight. After washing three times by TBST buffer, the membranes were incubated with horseradish peroxidase (HRP)‐conjugated secondary antibody targeted against rabbit or mouse for 1 hour at room temperature. Immunoblots were detected by an enhanced chemiluminescence kit (ECL, Pierce). The results of ECL were calculated by the software Image *J*.

### 2.11 Immunohistochemistry

After being fixed with 4% paraformaldehyde for 10 minutes, and incubated in 0.3% Triton X‐100 solution for 10 minutes, brain sections underwent antigen retrieval by citrate antigen retrieval solution and blocked with 1% bovine serum albumin (BSA) for 1 hour. Then, the sections were incubated with primary antibodies anti‐P2Y6 receptor (1:50, Alomone), Iba1 (1:200, Novusbio), GFAP (1:200, Millipore), Tuj‐1 (1:200, Millipore), NeuN (1:200, Millipore), or Lamp‐2 (1:200, Millipore) overnight at 4°C. After three five minutes times washes in PBS, sections were incubated with fluorescent conjugated secondary antibodies for 1 hour at 37°C. The sections were observed under a TCS SP5 Confocal Scanning System (Leica). TUNEL staining (TdT‐mediated dUTP Nick End Labeling) was performed for apoptotic analysis, by using One Step TUNEL Apoptosis Assay Kit (Meilun Biotechnology Co., Ltd).

Cells were treated with 100% methanol (chilled at −20°C) for 5 minutes. They were subsequently washed with ice‐cold PBS and incubated with 1% BSA in PBS for 60 minutes to block unspecific binding. Then, cells were incubated in the primary antibody anti‐MLCK (1:100, Abcam), Iba‐1 (1:200, Novusbio) overnight at 4°C. After three times washes by PBS, cells were incubated with the secondary antibody for 1 hour at 37°C in the dark. These cells were imaged with laser scanning confocal microscopy (Leica).

### 2.12 Real‐time PCR

Total RNA was isolated by Trizol reagent (Invitrogen) according to the previous manufacturer's protocols. Total RNA concentration was examined by a spectrophotometer (NanoDrop 1000, Thermo). 700 ng RNA was used for reverse transcription to make cDNA using a cDNA Synthesis Kit (Yeasen). A two‐step RT‐PCR cycling condition using qPCR SYBR Green Master Mix (Yeasen, Shanghai, China) was performed: melting step (95°C for 30 seconds), annealing step (40 cycles of 95°C for 10 seconds), and elongation (60°C for 30 seconds). mRNA level was normalized to GAPDH and was calculated by the 2^−ΔCt^ method.

Sequence primers are listed in the following table:

**Table:** 

Gene name	Forward primer (5′–3′)	Reverse primer (5′–3′)
IL‐1α	F:5′‐TCGGCAAAGAAATCAAGATG‐3′	R:5′‐ATGGTCAATGGCAGAACTGTAG‐3′
IL‐1β	F:5′‐TACATCAGCACCTCACAAGC‐3′	R:5′‐AGAAACAGTCCAGCCCATACT‐3′
IL‐6	F:5′‐TGATGCACTTGCAGAAAACAA‐3′	R:5′‐GGTCTTGGTCCTTAGCCACTC‐3′
IL‐10	F:5′‐GCGCTGTCATCGATTTCTCC‐3′	R:5′‐TGGCCTTGTAGACACCTTGG‐3′
TNF‐α	F:5′‐ACCCTCACACTCAGATCATCTT‐3′	R:5′‐GGTTGTCTTTGAGATCCATGC‐3′
TGF‐β	F:5′‐CACCGGAGAGCCCTGGATA‐3′	R:5′‐TGTACAGCTGCCGCACACA‐3′
GAPDH	F:5′‐AAATGGTGAAGGTCGGTGTG‐3′	R:5′‐AGGTCAATGAAGGGGTCGTT‐3′
P2Y6 receptor	F:5′‐GCAGCAAGGCGGCTCGTATG‐3′	R:5′‐TAGGCAGCAGCGAAGGTCTCC‐3′

### 2.13 Statistical analysis

Data are presented as mean ± SEM. For comparison between two groups, statistical significance was examined through Student's *t* test. For comparison among multiple groups, statistical significance between each group were examined by one‐way ANOVA followed by a Bonferroni correction for multiple analyses using GraphPad Prism 5 software. A value of *P* < .05 was considered significant.

## 3 RESULTS

### 3.1 P2Y6 receptor expression dramatically increased within 3 days after tMCAO

**Figure 1:**
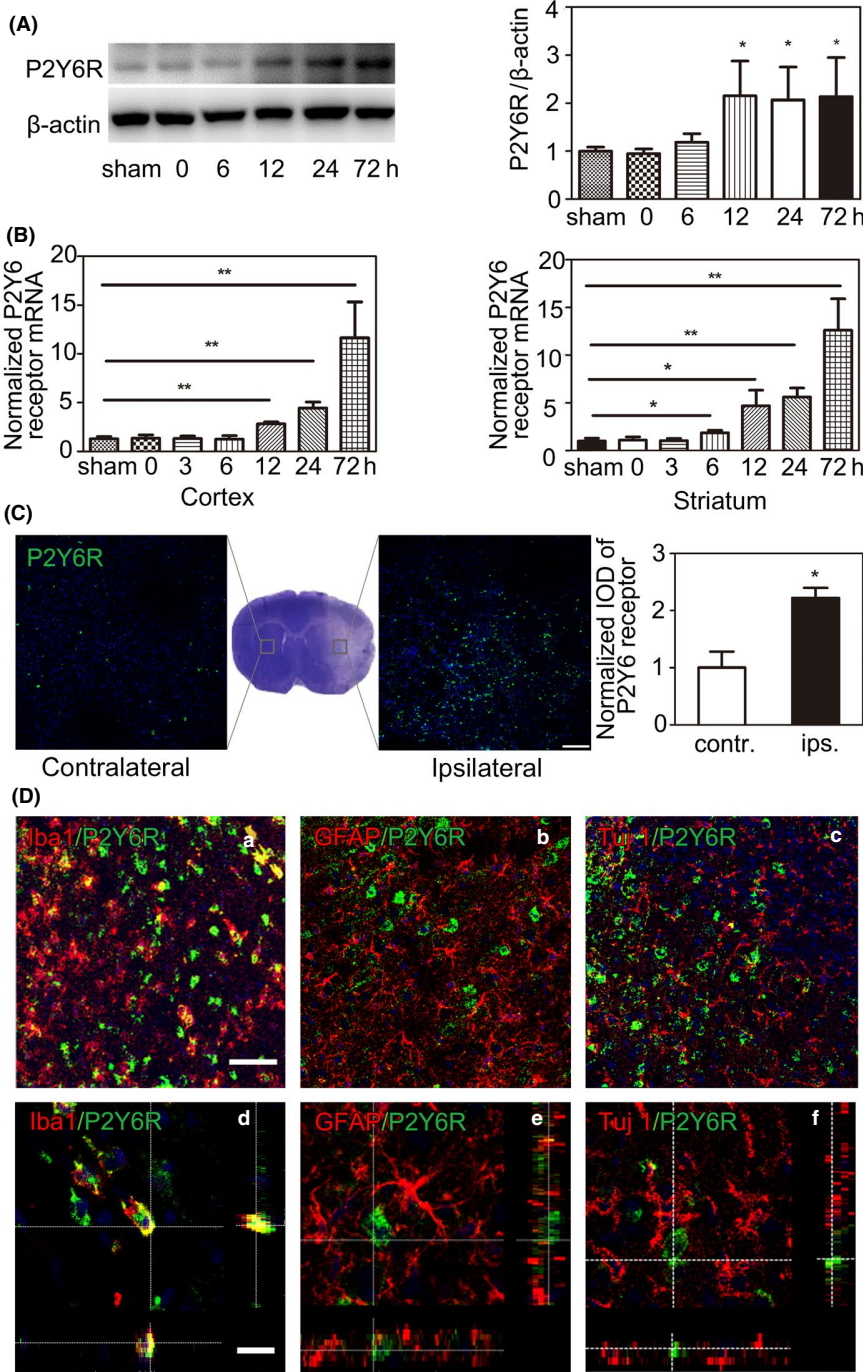
P2Y6 receptor expressed on microglia and dramatically increased within 3 d after tMCAO. A, Western blot and the quantification of P2Y6 receptor at 0 h, 6 h, 12 h, 1, and 3 d after ischemic stroke. B, Quantitative of RT‐PCR analysis for the expression of P2Y6 receptor at 0 h, 6 h, 12 h, 1, and 3 d after tMCAO. C, Representative fluorescence images of P2Y6 receptor in the contralateral and ipsilateral hemispheres of mice after tMCAO. Scale bar = 100 μm. D, Z‐stack for representative fluorescence images of (a) P2Y6 receptor (green) and Iba1 (red), (b) P2Y6 receptor (green) and GFAP (red), and (c) P2Y6 receptor (green) and Tuj 1 (red). Scale bar = 10 μm. Low magnification images are also presented respectively. Scale bar = 50 μm. Data were presented as mean ± SEM (n = 3 per group). *P < .05, **P < .01

To verify the expression of the P2Y6 receptor after ischemic stroke, we used Western blotting and RT‐PCR analysis. The results showed that the P2Y6 receptor protein level dramatically increased after tMCAO in ischemic mice. The increase began at 12 hours after tMCAO and maintained elevated for 3 days (Figure 1A). mRNA level of the P2Y6 receptor examined by RT‐PCR also increased after ischemia. P2Y6 receptor mRNA expression began to increase at 6 hours in the striatum and at 12 hours in the cortex. This increased expression maintained for 3 days (Figure 1B). We performed immunofluorescence staining of P2Y6 receptor in the contralateral and ipsilateral hemispheres of the mouse brain at 3 days after tMCAO. The fluorescence intensity of the P2Y6 receptor increased in ipsilateral hemisphere compared with contralateral hemisphere after ischemia (Figure 1C). The result indicated that ischemic stroke increased P2Y6 receptor expression in the brain within 3 days after tMCAO. To determine the cellular location of the P2Y6 receptor, we performed P2Y6R/ Iba1, P2Y6R/ GFAP, and P2Y6R/ Tuj‐1 double immunofluorescence staining in the peri‐infarct region at 3 days of tMCAO. The results showed that the P2Y6 receptor was mainly expressed in microglia, but not in GFAP^+^ astrocytes or Tuj‐1^+^ neurons in the peri‐infarct region of brain slice after tMCAO (Figure 1D).

### 3.2 P2Y6 receptor‐specific inhibitor MRS2578 blocked the phagocytosis of primary microglia under LPS and UDP stimulation

**Figure 2:**
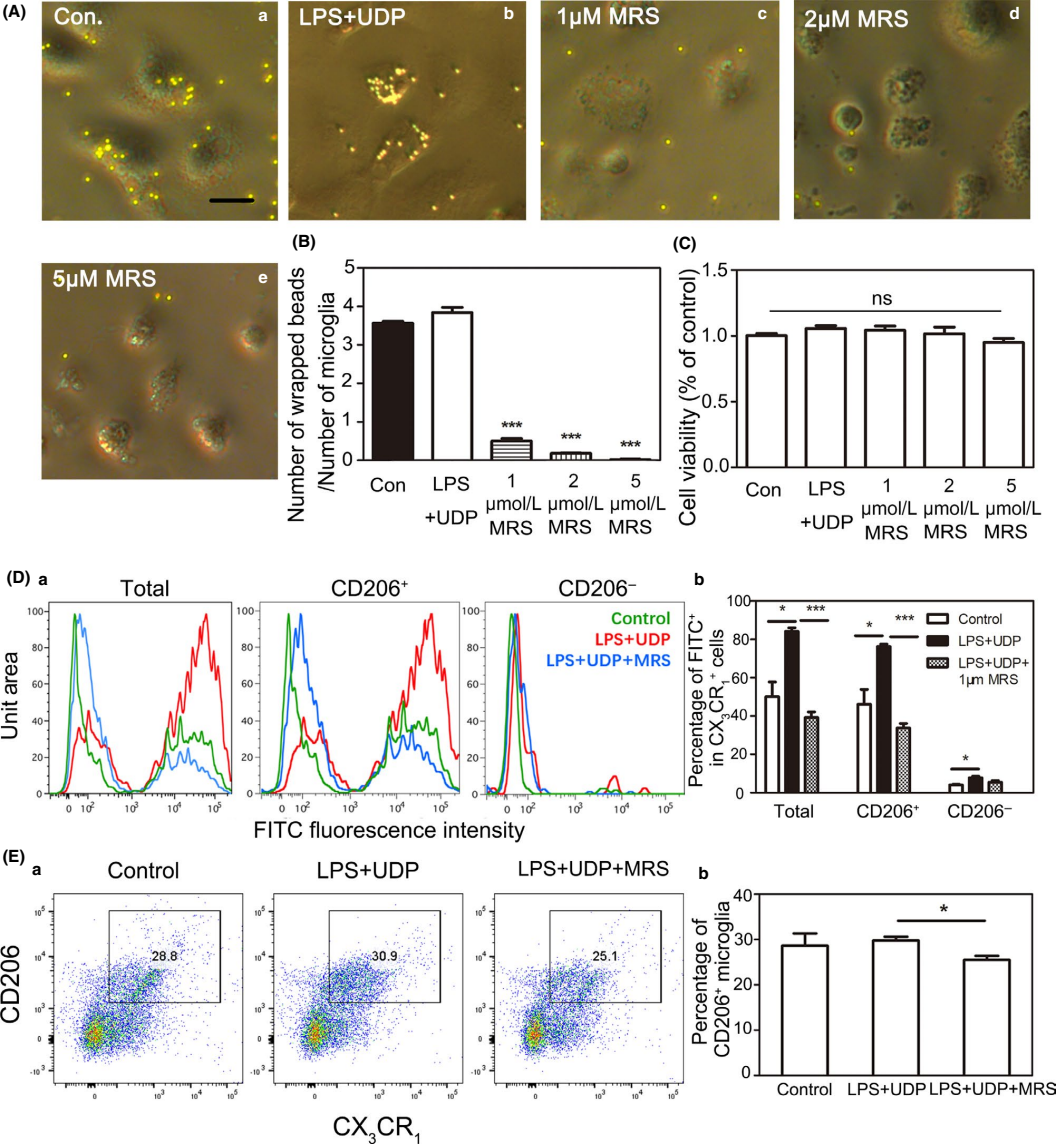
P2Y6 receptor‐specific inhibitor MRS2578 blocked the phagocytosis of primary microglia under LPS & UDP stimulation. A, Representative images of fluorescent latex beads (yellow) phagocytized by primary microglia under 12 h LPS (200 ng/mL) & UDP (100 μmol/L) stimulation in the control, LPS+UDP, 1 μmol/L MRS, 2 μmol/L MRS, and 5 μmol/L MRS group. (a) Control group, only cultured by basic culture medium of microglia, (b) MRS2578‐free group, 200 ng/mL LPS, and 100 μmol/L UDP in basic culture medium of microglia, (c) 1 μmol/L MRS2578 group, 200 ng/mL LPS, 100 μmol/L UDP, and 1 μmol/L MRS2578 in basic culture medium of microglia, (d) 2 μmol/L MRS2578 group, 200 ng/mL LPS, 100 μmol/L UDP, and 2 μmol/L MRS2578 in basic culture medium of microglia, (e) 5 μmol/L MRS2578 group, 200 ng/mL LPS, 100 μmol/L UDP, and 5 μmol/L MRS2578 in basic culture medium of microglia. Scale bar = 10 μm. B, Quantification for the number of beads per microglia under LPS (200 ng/mL) & UDP (100 μmol/L) stimulation (n = 3 per group). C, CCK‐8 assay in primary microglia without or with different concentrations MRS2578 under LPS+UDP stimulation for 12 h. D, (a) Representative flow cytometry analysis of microglia phagocytosis. Phagocytosis of fluorescein isothiocyanate (FITC)‐labeled latex beads by CD206^+^ and CD206^‐^ microglia. (Green, untreated control; Red, LPS+UDP treatment; Blue: LPS+UDP+1 μmol/L MRS2578 treatment); (b) Percentage of FITC^+^ cells in CX_3_CR_1_^+^ cells for total, CD206+, and CD206‐ microglia. E, (a) Representative diagrams of CD206^+^/CX_3_CR_1_^+^ cells in the control, LPS+UDP, LPS+UDP+MRS2578 group, (b) Percentage of CD206^+^/CX_3_CR_1_^+^ in the control, LPS+UDP, LPS+UDP+MRS2578 group. Data were presented as mean ± SEM. n = 3 per group. *P < .05, **P < .01, ***P < .001

The P2Y6 receptor is a purinoceptor member of P2receptor family as well as responsible for microglial phagocytosis in the CNS.^18^ To directly examine the inhibitory effect of MRS2578 on microglia in vitro, we cultured primary microglia using LPS and UDP stimulation to mimic the damage after ischemic stroke in vivo.^18^ After 12 hours LPS and UDP (100 μmol/L) stimulation, microglia were incubated with fluorescent latex beads for 2 hours. The results showed that the number of beads per microglia increased in the LPS+UDP group compared with the control group, and MRS2578 pretreatment blocked the increase induced by LPS+UDP stimulation (Figure 2A,B). The inhibitory effect was concentration‐dependent. In order to make sure if the reduction of the intake of latex beads was due to cell death, we tested cell viability after different concentrations of MRS2578 treatment by CCK8 assay. We chose 1, 2, and 5 μmol/L concentrations for MRS2578 treatment. The result showed that there was no significant difference between groups, indicating that the reduced intake of latex beads was due to inhibited phagocytosis, but not cell death (Figure 2C).

Furthermore, microglial phagocytosis was also assessed by FACScan analysis after MRS2578 treatment in vitro. We quantified the phagocytic efficiency by counting how many cells had phagocytosed fluorescent beads. The results showed 84.1% of total cells were positive for FITC in the LPS+UDP group, whereas 39.2% of total cells were positive for FITC in the LPS+UDP+MRS2578 group. We also demonstrated that the majority of phagocytic cells were CD206^+^. The percentage of CD206^+^ microglia decreased after MRS2578 treatment. Thus, we confirmed MRS2578 could inhibit the microglial phagocytosis in primary culture (Figure 2D,E).

### 3.3 Inhibiting P2Y6 receptor activity reduced the phagocytic function of microglia after tMCAO

**Figure 3:**
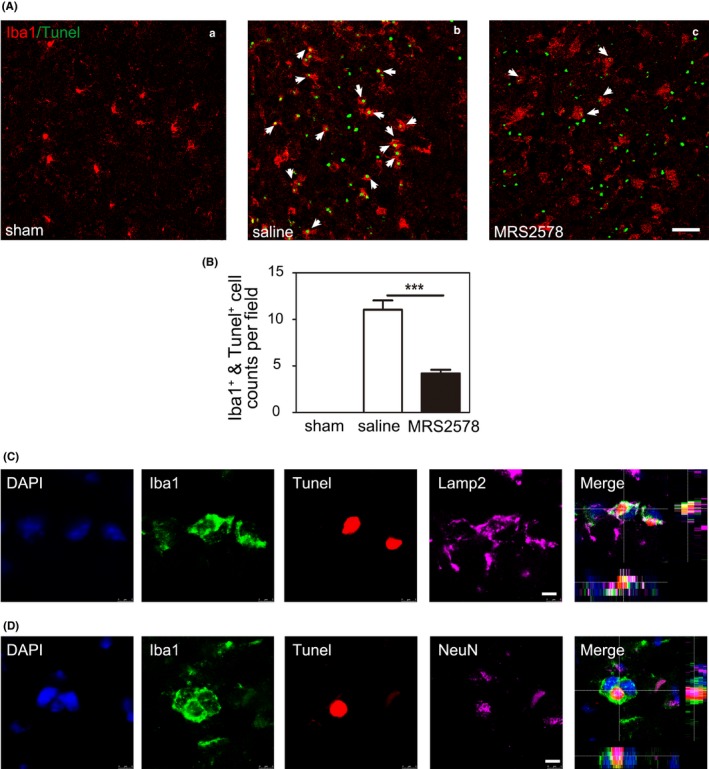
Inhibition of P2Y6 receptor activity reduced the phagocytic function of microglia after tMCAO. A, Representative fluorescence images of Iba1^+^ microglia (red) and TUNEL^+^ apoptotic cells (green) in the peri‐infarct region of brain slice in the sham (a), saline (b), and MRS2578 (c) group at 3 d after tMCAO. Scale bar = 50 μm. White arrows indicated Iba1^+^ cells engulfed TUNEL^+^ cells in the saline and MRS2578 group. B, Semiquantitative analysis of Iba1^+^ microglial phagocytosis for TUNEL^+^ cells. n = 4. C, Representative fluorescence images of Iba1^+^ microglia (green), lysosome marker LAMP‐2 (purple), and TUNEL^+^ apoptotic cells (red) in the peri‐infarct region of brain slice after tMCAO. 3D scanning with depth indicated Iba1^+^ cells phagocytosed TUNEL^+^ cells and transferred them into lysosomes. Scale bar = 5 μm. D, Representative fluorescence image of Iba1^+^ microglia (green), NeuN^+^ neurons (purple), and TUNEL^+^ apoptotic cells (red) in the peri‐infarct region of brain slice after tMCAO. Scale bar = 5 μm. 3D scanning with depth indicated Iba1^+^ cells engulfed TUNEL^+^ neurons

We explored whether the stroke‐induced microglial phagocytosis was mediated by the P2Y6 receptor in vivo. P2Y6 receptor activity was blocked by the selective P2Y6R antagonist MRS2578 (3 mg/kg) administered by intraperitoneal injection after three hours of tMCAO. The injection time point was decided by the RT‐PCR result. P2Y6 receptor significant increase since the time point. After three consecutive days of injections, the mice were sacrificed and the brains were collected. Iba1^+^ microglia and TUNEL^+^ apoptotic cells in the peri‐infarct region decreased in the MRS2578 group compared with the saline group. This suggested that the pharmacological inhibitor of the P2Y6 receptor MRS2578 significantly inhibited the microglial phagocytosis in the brain after tMCAO (Figure 3A,B). To further verify the conclusion, we performed Iba1/NeuN/TUNEL and Iba1/Lamp‐2/TUNEL immunostaining. The results showed that the TUNEL positive apoptotic neurons were localized to microglial lysosomes (Figure 3C,D).

### 3.4 Inhibition of P2Y6 receptor activity exacerbated neurological function deficit and brain injury after tMCAO

**Figure 4:**
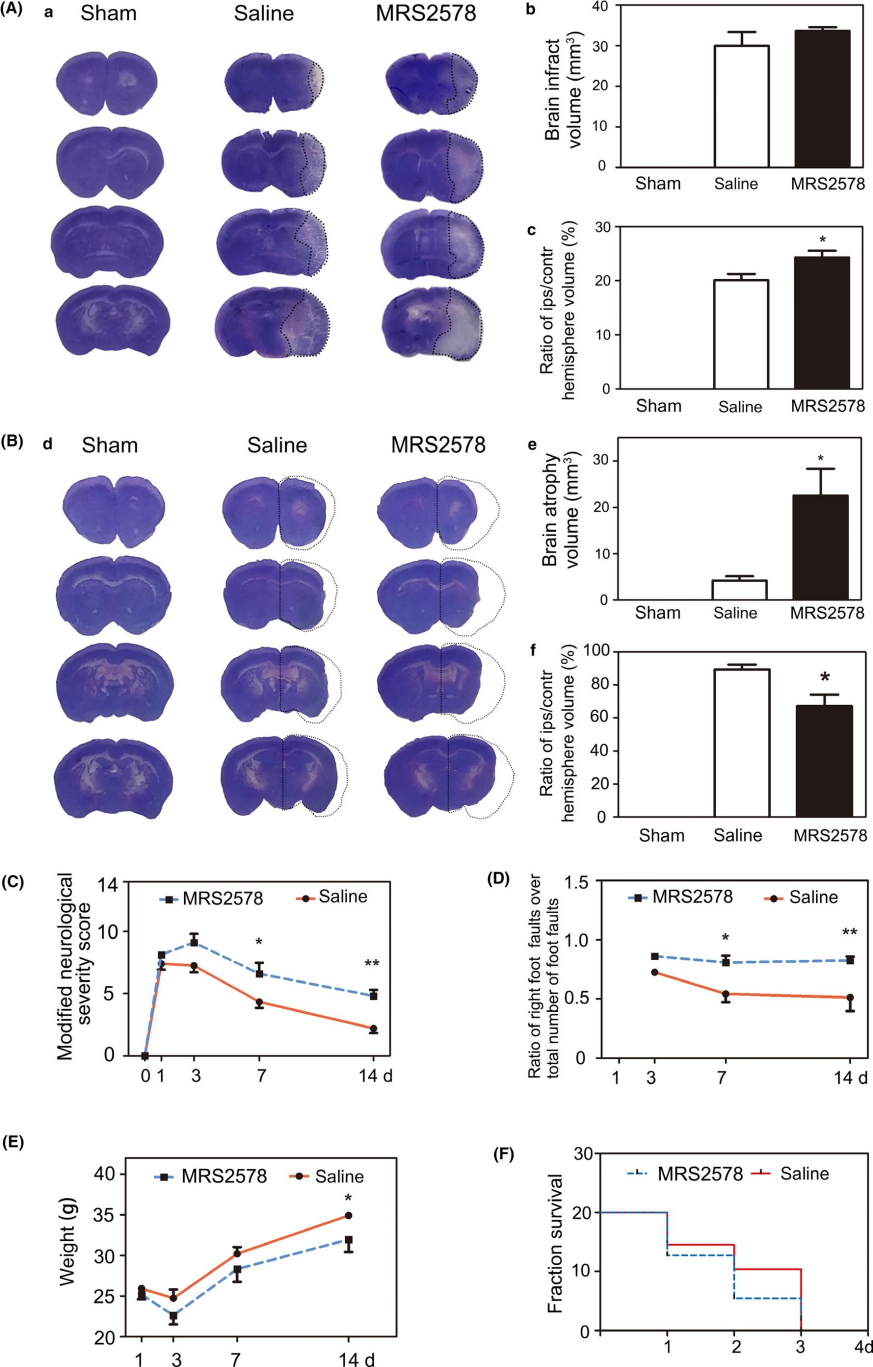
Inhibition of P2Y6 receptor activity exacerbated neurological function deficit and brain injury after tMCAO in mice. A, (a) Representative images of cresyl violet staining for brain infarct volume in the sham, saline, and MRS2578 group at 3 d after tMCAO. (b) Quantification of brain infarct volume in the sham, saline, and MRS2578 group at 3 d after tMCAO. (c) Quantification of the percentage of ipsilateral hemisphere volume/contralateral hemisphere volume in the sham, saline, and MRS2578 group at 3 d after tMCAO. B, (d) Representative images of cresyl violet staining for brain atrophy volume in the sham, saline, and MRS2578 group at 14 d after tMCAO. (e) Quantification of brain atrophy volume in the sham, saline, and MRS2578 group at 14 d after tMCAO. (f) Quantification of the percentage of ipsilateral hemisphere volume/contralateral hemisphere volume in the sham, saline, and MRS2578 group at 14 d after tMCAO. C. mNSS in the saline and MRS2578 group at 1, 3, 7, and 14 d after tMCAO. mNSS = modified neurological severity scores. D, Grid walking test in the saline and MRS2578 group at 3, 7, and 14 d after tMCAO. E, Body weight in the saline and MRS2578 group at 1, 3, 7, and 14 d after tMCAO. F, Survival curves of the saline and MRS2578 group within 3 d after tMCAO. Data were presented as mean ± SEM. n = 6‐11 per group. *P < .05, **P < .01

To analyze the effects of the inhibition of the P2Y6 receptor activity on tissue repair at the acute stage, we measured the brain infarct and atrophy volume at 3 and 14 days after tMCAO. There was a significant increase in infarct size at 3 days (Figure 4A), and in brain atrophy volume at 14 days after tMCAO in the MRS2578 group compared with the saline group (Figure 4B).

We next tested the neurological function in the saline and MRS2578 group. The mNSS system was used to assess neurological deficits at 1, 3, 7, and 14 days after tMCAO. The results showed that neurological scores were higher at 7 and 14 days in the MRS2578 group than the saline group (Figure 4C). Inhibition of P2Y6 receptor activity by MRS2578 increased the ratio of right foot fault at 3, 7, 14 days, which suggested ipsilateral limb suffered more severe damage in the MRS2578 group compared with the saline group (Figure 4D). There was a record of the two groups' body weight during the 14‐day period after tMCAO. The body weight decreased in the MRS2578 group compared with the saline group, especially at 14 days after tMCAO (Figure 4E). We also found that the death rate was significantly higher in the MRS2578 group compared with the saline group, which suggested the inhibition of microglial phagocytosis induced harmful effects after tMCAO (Figure 4F).

### 3.5 Inhibiting the P2Y6 receptor activity had no effect on the expression of inflammatory cytokines and neutrophil infiltration

**Figure 5:**
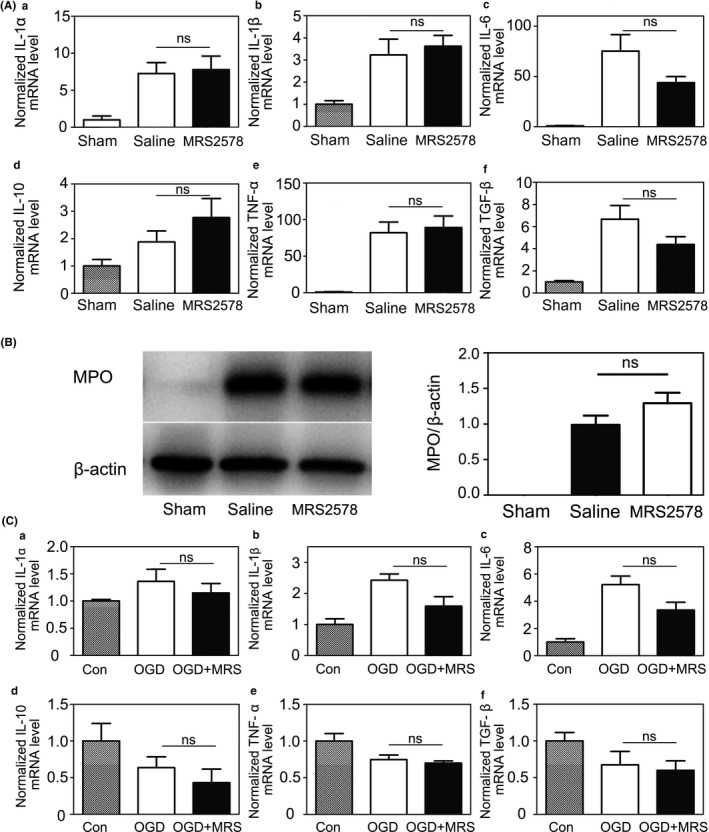
Inhibiting the P2Y6 receptor activity had no effect on the expression of inflammatory cytokines and neutrophil infiltration. A, Quantification of RT‐PCR analysis for the expression of IL‐1α (a), IL‐1β (b), IL‐6 (c), IL‐10 (d), TNF‐α (e), and TGF‐β (f) at 3 d after tMCAO in the sham, saline, and MRS2578 group. The data in sham group were normalized to 1. B, Western blot for the expression of MPO in the brain of sham, saline, and MRS2578 groups at 3 d after tMCAO (g). Quantification of the intensity ratios of MPO/ β‐actin (h) in the sham, saline, and MRS2578 group. The data in the saline group were normalized to 1. C, Quantification of RT‐PCR analysis for the expression of IL‐1α (a), IL‐1β (b), IL‐6 (c), IL‐10(d), TNF‐α (e), and TGF‐β (f) after 1 h OGD and 11 h reoxygenation in the control, OGD and OGD+MRS2578 group. The data in the control group were normalized to 1. Data were presented as mean ± SEM. n = 3‐4 per group. *P < .05, **P < .01

To determine whether inhibition of microglial phagocytosis by MRS2578 affected the expression of inflammatory cytokines in the brain after tMCAO, we further examined mRNA expression of IL‐1α, IL‐1β, IL‐6, IL‐10, TNF‐α, and TGF‐β. The results showed that mRNA level of IL‐1α, IL‐1β, IL‐6, IL‐10, TNF‐α, and TGF‐β upregulated after 3 days of tMCAO, except IL‐10 (Figure 5A). However, the expression of these six inflammatory cytokines had no significant differences between the MRS2578 group and the saline group at 3 days after tMCAO.

In order to investigate neutrophil infiltration at the acute stage after tMCAO, we detected MPO expression using Western blotting at 3 days after tMCAO (Figure 5B). The result showed no significant difference between the saline and the MRS2578 group, which suggested that MRS2578 had no effect on neutrophil infiltration after tMCAO.

In vitro, we also performed real‐time quantitative PCR to examine the inflammatory factor expression after 1 hour of OGD and 11 hours of reoxygenation in the coculture of microglia and neurons. The results showed the increase of IL‐1α, IL‐1β, and IL‐6 expression, and no change of IL‐10, TNF‐α, and TGF‐β expression. We found no significant difference in cytokine expression between the OGD and OGD+MRS2578 groups (Figure 5C). The results indicated that the deterioration of neurological function and brain injury were caused by the inhibition of microglial phagocytosis rather than the expression of inflammatory cytokines and neutrophil infiltration.

### 3.6 P2Y6 receptor inhibition affected MLCK expression in the ischemic brain and cultured microglia

**Figure 6:**
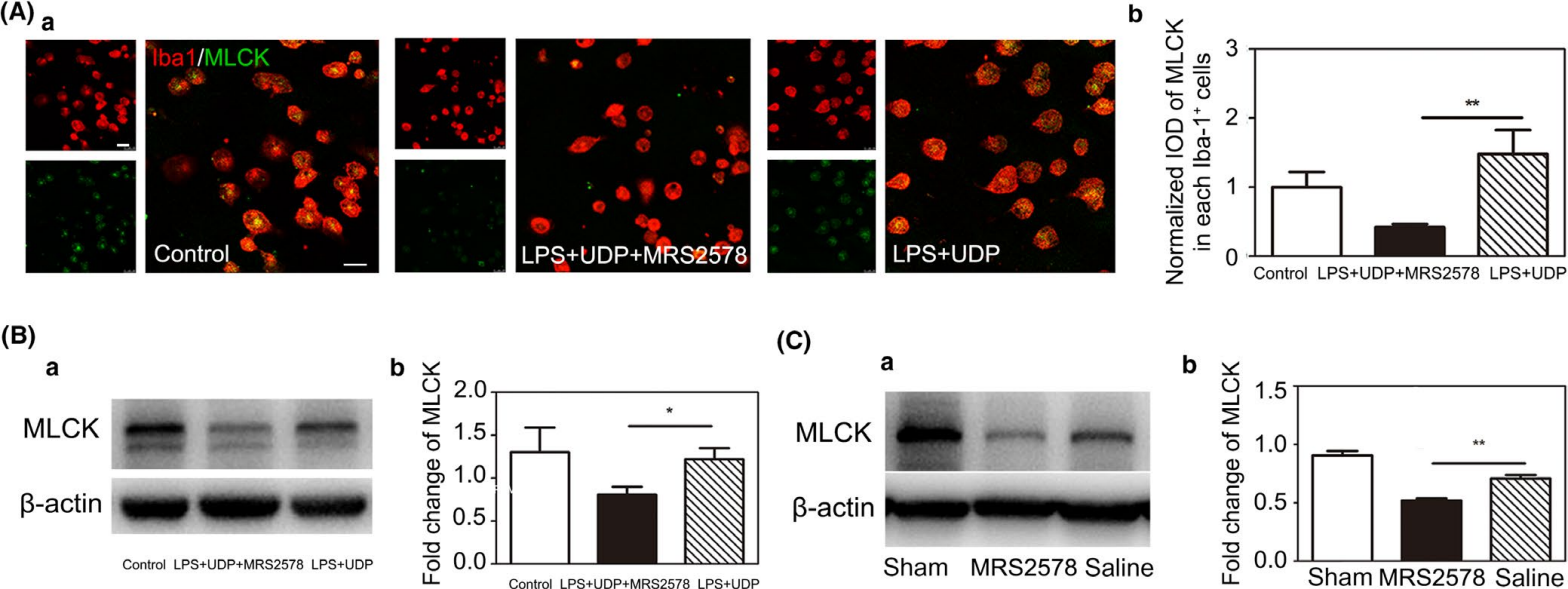
P2Y6 receptor antagonist MRS2578 inhibit MLCK expression in mice at 3 d after tMCAO and cultured microglia. A, (a) Representative fluorescence images of MLCK in Iba‐1^+^ cultured primary microglia. Scale bar = 25 μm. (b) Quantitative analysis of MLCK in each Iba1^+^ microglia. n = 4 per group. B, (a) Western blot for the expression of MLCK in the control, LPS+UDP and LPD+UDP+MRS2578 group. (b) Quantification for the intensity ratios of MLCK/β‐actin in the control, LPS+UDP and LPD+UDP+MRS2578 group. C, (a) Western blot for the expression of MLCK in the sham, saline, and MRS2578 group at 3 d after tMCAO. (b) Quantification for the intensity ratios of MLCK/β‐actin in the sham, saline, and MRS2578 group. Data were presented as mean ± SEM. n = 3‐4 per group. *P < .05, **P < .01

Since the formation of the phagocytic cup requires cytoskeletal remodeling and that MLCK modulates cytoskeletal morphology, we investigated MLCK function in P2Y6 receptor‐mediated microglial phagocytosis.^29^, ^30^ Western blotting was used to examine MLCK expression at 3 days of tMCAO. Results showed that MLCK expression was down‐regulated in the MRS2578 group compared with the saline group after tMCAO. The results suggested that the cytoskeletal MLCK could participate in the P2Y6 receptor‐mediated microglial phagocytosis after tMCAO at the acute stage (Figure 6B).

Since MLCK is expressed in many types of cells in addition to microglia,^31^, ^32^ we performed in vitro immunostaining to address whether MLCK decreased in microglia specifically. Primary microglia were activated by LPS+UDP treatment. The double immunostaining of Iba‐1/MLCK and Western blotting of MLCK results showed that MLCK expression decreased in microglia after MRS2578 treatment (Figure 6A,C).

## 4 DISCUSSION

To better understand the role of microglial phagocytosis in neurological recovery after ischemic stroke, we used MRS2578 to block the P2Y6 receptor after tMCAO. We found that P2Y6 receptor‐mediated microglial phagocytosis benefited neurological recovery after tMCAO, and MLCK was involved in the pathway of P2Y6 receptor‐mediated microglial phagocytosis.

After focal brain ischemia, the initial phagocytic response is mainly performed by microglia.^33^ Phagocytic microglia rapidly reach their maximum number as early as day 1 and stayed at the same level in the following 3 days after ischemic stroke.^34^ This suggested that the debris clearance mediated by microglia happens at the acute stage after brain injury. Therefore, we studied the P2Y6 receptor expression and microglial phagocytosis within 3 days after tMCAO. P2Y6 receptor expression increased in kainic acid‐treated rats,^18^ and our results supported that the mRNA and protein level of P2Y6 receptor expression increased at the acute stage after tMCAO.

Increasing evidence show that microglial phagocytosis plays a canonical role in neurological recovery after brain injury.^13^, ^14^, ^15^, ^35^ Several pathways are involved in microglial phagocytosis after brain injury, including CD36, MerTK, MFG‐E8, TL4, and TREM2.^13^, ^14^, ^15^, ^36^ It is thought that microglial phagocytosis plays a positive role in debris clearance and reconstruction of neuronal networks in brain pathologies such as MS, ALS, trauma.^37^, ^38^, ^39^ Ischemic stroke causes massive cells death in the brain, and microglial phagocytosis is involved in the clearance of dead cell and debris. Studies showed that brain injury aggravated after inhibiting microglial phagocytosis through CD36 or TREM2 in ischemic stroke.^13^, ^14^ The upregulated P2Y6 receptor might mediate microglial phagocytosis to clean dead cells and dangerous debris after kainic acid‐induced injury.^18^ P2Y6 receptor also played role in cleaning myelin debris and apoptotic neurons post 14 days after radiation‐induced brain.^19^ In 3‐4 months old Alzheimer's disease model, microglia lost the function of cleaning amyloid deposition, which was associated with P2Y6 receptor.^40^ Antagonist MRS2578 inhibited the P2Y6 activity specifically.^41^ Our results indicated that the cleaning function of microglia decreased after inhibiting the P2Y6 receptor‐mediated microglial phagocytosis by MRS2578, which aggravated ischemic brain injury in the acute stage. Briefly, first, the expression of P2Y6 receptor increased after tMCAO. Second, P2Y6 receptor mainly expressed on microglia in the brain. Third, P2Y6 receptor antagonist MRS2578 blocked microglial phagocytosis. Fourth, brain atrophy and edema volume increased after MRS2578 treatment. Fifth, MRS2578 treatment exacerbated the results of mNSS and Grid walking test after tMCAO.

The proportion of M2 phenotype microglia may affect phagocytosis.^42^ In CNS activated M1 microglia and macrophages release proinflammatory mediators, such as TNF, IL‐1, and IL‐6. Activated M2 microglia and macrophages mainly clear cellular debris through phagocytosis and release numerous protective and trophic factors.^43^, ^44^ Our FACScan analysis showed that M2 microglia mainly played a phagocytic role under LPS and UDP stimulation. This supports the notion that the predominantly phagocytic microglia are M2 phenotype. In various models of CNS injury, the majority of newly recruited microglia and macrophages at the site of injury are M2 phenotype, while cells expressing M1 genes are predominately present about 1 week after injury.^45^, ^46^ Although M1 microglia could be stimulated by LPS or IFN‐γ,^47^ the proportion of M2 microglia in our study did not decrease after LPS+UDP stimulation, which might due to the addition of P2Y6 receptor agonist UDP. Meanwhile, our result showed that MRS2578 treatment reduced the proportion of M2 phenotype microglia, which decreased phagocytosis. In our study, the worsen outcome of ischemic mice may be due to the reduction of M2 microglia. The balance between different microglial phenotypes could affect regenerative therapies after CNS injury.^43^, ^44^, ^48^, ^49^ Increasing evidence show that microglia polarize into a variety of phenotypes at different stages of injuries in CNS.^50^, ^51^ Decreased expression of Arg1 in STAT6^−/−^ microglia/macrophages led to impairments in phagocytosis and loss of anti‐inflammatory modality which indicates that microglia phenotype is closely related to phagocytosis.^52^ Direct evidence for the relationship between specific microglia phenotype and phagocytosis should be investigated in the future.

Inflammation also affects the neurological function after injury.^53^ Microglia and macrophages are immune cells in the brain and play a role in the inflammation response after brain injury.^3^, ^50^, ^54^ In response to brain injury, microglia promptly acquire properties of reactive species generation, antigen presentation, phagocytosis, and the production of inflammatory mediators including IL‐1, TNF‐α, and IL‐6.^55^ Proinflammatory cytokines released by necrotic cells induce subsequent neutrophil infiltration to ischemic tissue.^56^We found that inflammatory cytokine mRNA expression of IL‐1α, IL‐1β, IL‐6, IL‐10, TNF‐α, and TGF‐β both in vivo and in vitro, and infiltration of neutrophils in vivo did not change with MRS2578 treatment. This meant that MRS2578 treatment only changed the microglial phagocytic function without affecting inflammatory response. It is unclear whether inhibiting microglial phagocytosis certainly influences their secretion of inflammatory cytokines. The worsened brain injury after MRS2578 treatment could result from the deficiency of dead cells and tissue debris clearance rather than inflammatory cytokine levels and leukocyte infiltration.

Finally, MLCK was involved in P2Y6 receptor‐mediated microglial phagocytosis. Myosins cross‐bridge with actin filaments through MLCK and mediate contraction, which may be involved in phagocytosis.^31^, ^32^ Our results indicated that the inhibition of the P2Y6 receptor decreased MLCK expression in cultured microglia. The Rac1/MLCK signaling pathway is downstream of the P2Y6 signaling cascade in radiation injury model.^19^ Our result showed that the level of Rac1 expression did not differ between the MRS2578 and saline group, suggesting that Rac1 signaling was not involved in microglia phagocytosis after ischemic stroke (Figure S1). MLCK expression in the sham group was higher than that in the saline and MRS2578 groups, which may vary due to massive dead cells in the ischemic brain. Admittedly, it is reported that P2Y6 receptor also exists on other cell types,^57^, ^58^ and our results showed some P2Y6 receptor‐expressing cells are Iba1 negative (Figure 1D). In addition, both resident microglia and infiltrating macrophages are Iba1 positive. Therefore, other cell types and infiltrating macrophages may also play a role in the disease process. To further verify our conclusion, we will apply P2Y6R^flox/flox^::CX_3_CR_1_‐Cre mice and remove peripheral macrophages by irradiation which could provide direct evidence of the P2Y6 receptor‐mediated microglial phagocytosis in the future. Our study only blocked P2Y6 receptor‐mediated microglial phagocytosis at the acute stage of tMCAO, and the role of microglial phagocytosis at the late stage will be investigated in the future.

## 5 CONCLUSION

In conclusion, our study is the first evidence of P2Y6 receptor‐mediated microglial phagocytosis after tMCAO. We demonstrated that P2Y6 receptor inhibition increased brain infarct and atrophy volume, and aggravated neurological outcomes after tMCAO. The worsen outcome might be due to the loss of microglial phagocytosis. These novel findings suggest that P2Y6 receptor‐mediated microglial phagocytosis can be a potential therapeutic target after ischemic stroke.

## CONFLICT OF INTEREST

The authors declare that they have no competing interests.

## ETHICAL APPROVAL

Animal protocol was approved by the Institutional Animal Care and Use Committee (IACUC) of Shanghai Jiao Tong University, Shanghai, China. All animal procedures were performed to minimize the pain or discomfort in accordance with current protocols.

## Data Availability

All data generated or analyzed during this study are included in this published article.
