# Supplementary figures and images for: P2Y6 receptor inhibition aggravates ischemic brain injury by reducing microglial phagocytosis
Source: CNS Neurosci Ther. 2020 Mar 10;26(4):416–29. doi: 10.1111/cns.13296 (PMC7080436; doi:10.1111/cns.13296)

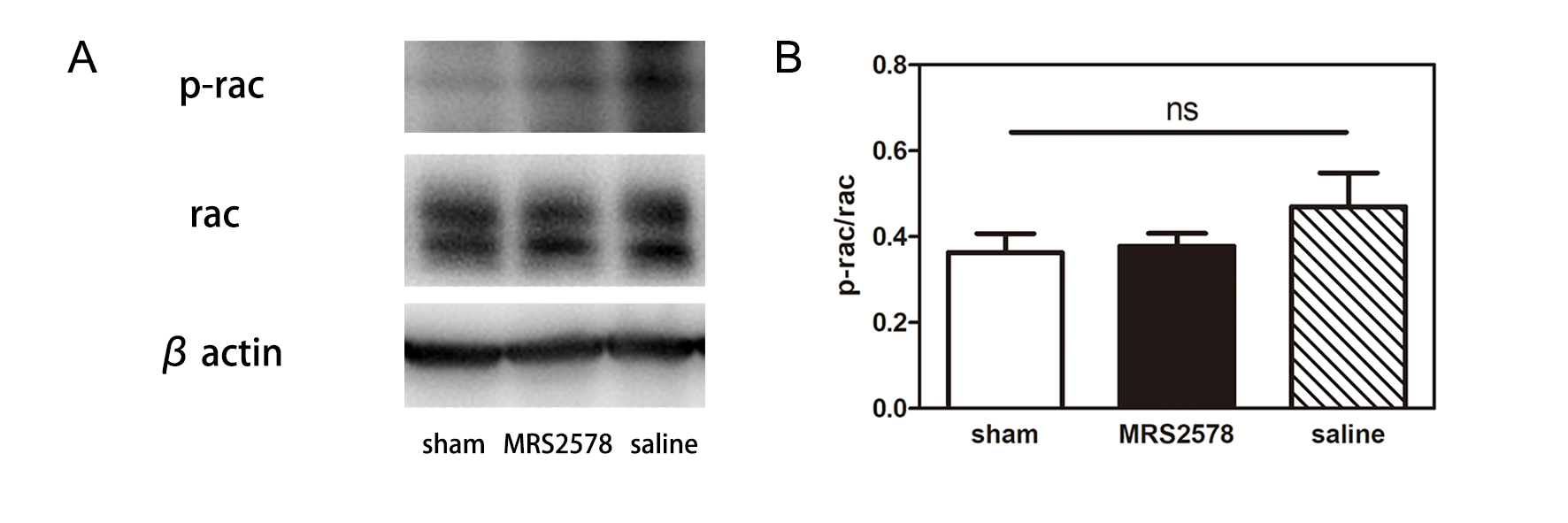

Supplement: Supplementary file 1 [file CNS-26-416-s001.tif]
